# A comprehensive performance analysis of advanced hybrid MPPT controllers for fuel cell systems

**DOI:** 10.1038/s41598-024-63074-z

**Published:** 2024-05-31

**Authors:** Ezzeddine Touti, Shaik Rafikiran, Besma Bechir Graba, Mouloud Aoudia, S. Senthilkumar

**Affiliations:** 1 Department of Electrical Engineering, College of Engineering, Northern Border University, 91431 Arar, Saudi Arabia; 2 Department of Electrical and Electronics Engineering, Sri Venkateswara College of Engineering, Tirupati, 517507 Andhra Pradesh India; 3 Department of Industrial Engineering, College of Engineering, Northern Border University, 91431 Arar, Saudi Arabia; 4 Department of ECE, E.G.S. Pillay Engineering College, Nagapattinam, Tamil Nadu 611002 India

**Keywords:** Ability to track MPP, Convergence time, Duty signal, Effectiveness of MPP tracking, Fuel stack, Good static performance, Plus load voltage oscillations, Engineering, Electrical and electronic engineering

## Abstract

The present power generation corporations are working on Renewable Power Systems (RPS) for supplying electrical power to the automotive power industries. There are several categories of RPSs available in the atmosphere. Among all of the RPSs, the most general power network used for Electric Vehicles (EVs) is hydrogen fuel which is available in nature. The H_2_ fuel is fed to the Proton Exchange Membrane Fuel Stack (PEMFS) for producing electricity for the EV stations. The advantages of this selected fuel system are more power conversion efficiency, environmentally friendly, low carbon emissions, more power density, less starting time, plus able to work at very low-temperature values. However, this fuel stack faces the issue of a nonlinear power density curve. Due to this nonlinear power supply from the fuel stack, the functioning point of the overall network changes from one position of the I–V curve to another position. So, the peak voltage extraction from the fuel stack is not possible. In this article, there are various metaheuristic optimization-based Maximum Power Point Tracking (MPPT) methodologies are studied along with the conventional methods for obtaining the Maximum Power Point (MPP) position of the PEMFS. From the simulative investigation, the Continuous Different Slope Value-based Cuckoo Search Method (CDSV with CSM) provides better efficiency with more output power. Also, for all the MPPT methods comprehensive analysis has been made by utilizing the simulation results.

## Introduction

The availability of conventional sources is very low. Also, it affects the atmospheric conditions by releasing hazardous gases in nature. Currently utilized conventional resources are oil, coal, uranium, natural gas, and steam power plants. In^1–4^, the researchers referred to the coal-fired power technology for giving electricity to the steel production industry. Also, the coal power concept is used for the cement industry and aluminum industry. However, the demerits of coal-fired power networks are excessive environmental problems, nonrenewable sources, highly destructive natural habits, more coal mining impact, more potential radioactive, plus displacements of human settlements. The uranium-dependent power networks are applied in the central grid power supply network to limit the problems of the coal systems. Nuclear energy originates from the splitting of uranium atoms by applying the fission process. This system produces heat which is transferred in the form of steam^5^. The available steam content is sent to the turbine chamber with high pressure for moving the generator. The nuclear system typically gives 37% functioning efficiency which is acceptable. However it produces high wastage and the possibility of linkages happening in the system^6^. So, the present researchers have started moving towards Renewable Sources (RS) which are differentiated based on their availability in nature.

From the current literature study, the RSs are bioenergy, wind, ocean energy, geothermal power, hydropower, solar power, and fuel stack power. Here, bioenergy is one of the diverse power sources and it helps at the critical peak load demand circumstances. In this power production network, biomass fuels are transferred into steam energy to obtain a useful power supply. Here, geothermal systems are applied for food dehydration, milk pasteurizing, plus gold mining. Also, this network is useful for building heating, homes, and fisheries applications^7^. However, the geothermal networks create high surface instability, plus more set-up development costs. Also, it creates underground water pollution and generates hazardous waste. The geothermal power network problems are compensated by using wind power technology. In^8^, the researchers discussed the wind power network which is mostly used for remotely located people's power consumption. Also, the wind power networks are utilized in the water pumping, electric livestock fencing, and lighting systems. However, the wind systems create noise issues and highly impact the wide life animals. In addition, wind power is unpredictable and less suitable for all regions on the earth.

The hydro energy supply networks produce the energy at a time to the grid, and it provides essential backup energy to the local loads under a sudden shortage of power supply conditions. This type of system provides many benefits beyond power production which are flood control, clean drinking water, and more useful for agricultural water supply^9^. Here, the water flow kinetic power is transformed into rotational power. These types of systems are utilized for small industry settings and construction purposes. However, the hydro networks are hidden and dangerous, more difficult to manufacture, excessive cost required for installation, plus very poor resistance to working fluid. Also, it needs more catchment area when associated with the thermal power supply networks. The disadvantages of hydro systems are compensated by selecting the sunlight networks. The sunlight systems are introduced in the eighteenth century itself. Significantly, sunlight cell production has been done by using silicon material^10–14^. However, the sunlight cells are implemented by considering the polycrystalline, monocrystalline, and less efficient thin-film methodology. Here, the solar strings are interlinked by selecting the monocrystalline.

**Figure 1:**
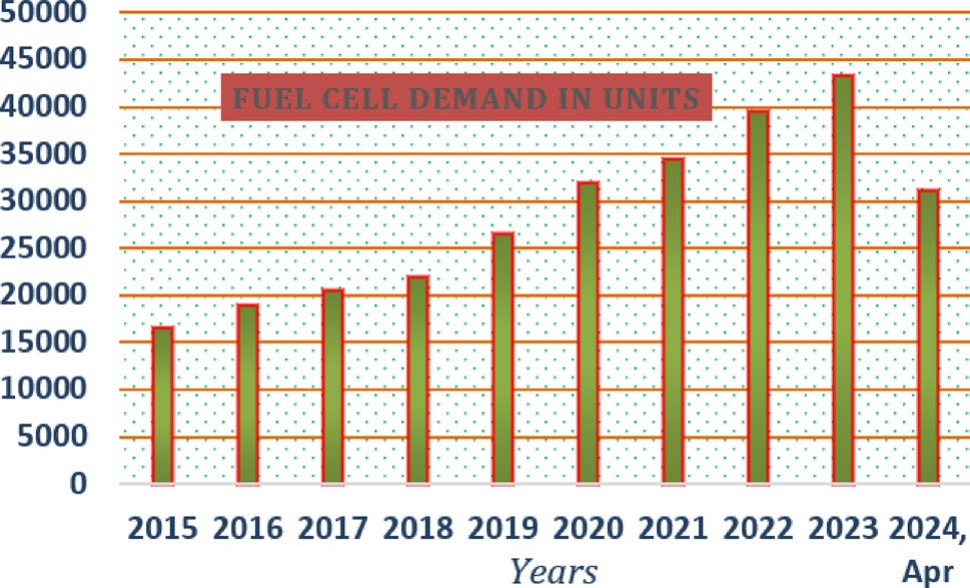
Statics of available fuel stack technologies.

The sunlight supplies energy to the external circuit like a diode. In this, the silica material collects a huge amount of sunlight insolation’s. After that, the available free electrons run with the help of sunlight energy intensity. Sunlight string is developed based on the series integration of PV modules. Here, the single-cell potential is 0.83 V which is low and may not be utilized for any application. So, the thousands of solar modules are combined to form the sunlight network. The sunlight system merits are flexible maintenance, low level of difficulty, less complications in design, low transportation price, and more reliability^15^. However, solar is more expensive, requires lots of space, and completely depends on the environmental conditions. Also, the solar panels are inefficient, and low life span. As a result, the solar energy is not efficient for the automotive networks. The fuel stack is a very promising technology for electric vehicle technology when associated with the sunlight network. The demand for this technology is illustrated in Fig. 1. The fuel stack technology was introduced in the seventeenth century, and it is a very rapidly growing technology for EV networks.

In^16^, the researchers studied the reversible fuel cells applied for sudden shortage energy applications. Also, it acts as a battery for charging the huge weight of battery packs. The advantages of this cell are zero-emission power, robust reliability, more efficiency, high scalability, and low implementation cost. However, this cell is less efficient when associated with other electrochemical accumulators. The Direct Methanol Fuel Cell (DMFC) is placed in the middle of the smart grid network and battery charging station for the spontaneous improvement of busbar voltage. Here, a methanol fluid acts as a membrane for conducting the chemical reactions of the DMFC. In this stack, the oxygen is combined with the methanol for the production of electrical energy. The major concern about this system is no need for reformers. So, the system size is very compact. Also, it has the property of high energy density and is available in the form of liquid under normal environmental conditions^17–20^. However, this system has the drawbacks of low working efficiency which is equal to 33%, and it involves a very sluggish anode reaction. These fuel network disadvantages are limited by replacing the alkaline cells to improve the current density of the microgrid network. This type of fuel system works at very low-quality H_2_ fuel with high power conversion efficiency^21^. The features of alkaline cells are the ability to function at below zero-degree centigrade temperature, and more possibility of using the nonnoble catalyst. Also, it produces energy with zero environmental pollution and provides robust reliability. However, this network size is more, and not useful for mobile charging applications. In this work, the PEMFC is designed by utilizing mathematical equations. The features of this system are compact design, fast startup time, and good energy density^22^.

The PEMFC supplies completely distorted nonlinear energy to the system. So, the MPPT block is interfaced with the source network for making the uniform power supply. In^23^, the authors reviewed the various categories of power point identifiers which are generally illustrated as soft computing, machine learning, swarm intelligence, fuzzy systems, and neuro intelligence. P&O is the one conventional concept that is frequently applied for all-renewable networks to make the MPP of the system at one position. This method features less cost of operation, is more flexible, anyone can handle the circuit, and it can be applied where the accuracy of the system is not needed. However, this method does not provide high accuracy at quick variation of the water membrane content of the fuel stack. So, the Modified Incremental Conductance (MIC) controller is implemented in the work^24^ for moving the operating point of the network from the local MPP region to the required peak power position. The manufacturing cost of MIC is more when associated with the P&O block. Also, it takes a high amount of time to identify the proper MPP location. As a result, these category controllers may not be useful for the fast changes in the fuel stack output temperature values.

The closed loop monitored I–V curve slope-dependent power point identifier is introduced in the article^25^ for obtaining the uniform output voltage of the quadrature converter. Here, the slope variation is observed for moving the fuel stack power from the source network to the battery pack. This controller collects the all-fuel stack parameters for maintaining the constant battery supply voltage. However, the cost of this closed-loop monitored network is higher. The incremental current density-based power point identifier is selected in^26^ for matching the fuel stack resistance with the consumer load impedance thereby enhancing the load stability of the consumer load. This technology applies only to the uniform water membrane conditions of the power system. In this manuscript, the continuous different slope value-based cuckoo search methodology is proposed for the rapid variation of the fuel power network temperature. This algorithm enhances the voltage profile of the overall system by optimizing the load current. The features of this concept are fast MPP identification, more robustness, the ability to function at any water membrane value of the fuel stack, and good operational efficiency. Also, this algorithm takes a very low convergence time value for obtaining the peak power from the PEMFC^27–31^.

Another drawback of the fuel stack is more amount of current generation which is not directly connected to any consumer load because of the high energy conduction losses. So, the power transformation technologies are used in^32,33^ for optimizing the load current consumption. In^34^, the researchers investigated the isolated technology for enhancing the renewable source voltage. All of the isolated power DC–DC circuits are manufactured by considering the one more rectifier, and single-phase transformer. So, the combination of transformers, and rectifier circuits along with the isolated converter increases the entire system's cost, and size. Moreover, it needs high-power electronics expertise candidates to run the entire power production network. So, the non-isolated technology-dependent power conversion concept is proposed in this work for reducing the current supply rating of the fuel stack. These converter topology features are more compact, have low passive components usage, low level of converter output power fluctuations, and easy operation.

## Literature review on power point tracking methods

From the present literature survey, most of the electric vehicles fed PEMFS give nonlinear voltage versus current characteristics. As a result, the peak voltage extraction from the source is very difficult. So, the MPPT controllers play a predominant role in the present electric vehicle industry. In^35^, the authors implemented the PSO-based P&O controller for tracking the MPP of the PEMFS. Here, at the starting stage of the MPPT controller operation, the P&O controller moves the functioning point of the fuel stack from the local MPP position to the global MPP position. Later, the PSO controller reduces the fluctuations across the MPP for extracting the peak power from the PEMFS. The merits of this hybrid P&O-PSO controller are less convergence time for tracking the MPP point of the fuel stack, good dynamic response, and easy handling^36^. However, its implementation cost is more when equated with the PSO controller.

To overcome the drawbacks of this hybrid controller, an ANN-based PSO controller is utilized in the hybrid wind/fuel stack power generation system for generating the switching pulses to the interleaved DC–DC converter. The selected input signals to the artificial neural network-based PSO controller are wind speed, generator voltages, fuel stack current, and fuel stack output stack voltage. Based on these input signals data, the multilayer perceptron neural network starts working and gives the voltage error signal to the PSO block to optimize the oscillations across the fuel stack MPP position^37^. The merits of this hybrid controller are the fast-tracking speed of MPP, quick static response, ease of handling, and fewer iterations required to find the required object. However, this controller gives continuous converter output voltage distortions under different water membrane conditions of the fuel stack.

In^38^, the authors used the fuzzy rule-based PSO power point tracking controller for identifying the functioning point of the PEMFS under various operating temperature conditions of the fuel stack. Here, the fuzzy rules are designed by utilizing the PSO controller. The features of this hybrid controller are fast convergence speed, a smaller number of sensors to sense the fuel stack parameters, plus high adaptability. However, this hybrid MPPT technique may not be suitable for the continuous variation of the fuel stack parameters such as hydrogen, water membrane volume, and functioning temperature of the fuel stack. So, the genetic algorithm-optimized fuzzy logic technology is used for handling the fuel stack at various input hydrogen decomposition constraints, and water membrane values. Here, the fuzzy working efficiency, and the MPP tracking accuracy of the fuel stack are improved by utilizing the genetic algorithm. In addition, the fuzzy MPPT controller gives high steady-state oscillations across the functioning point of the fuel stack which are optimized by utilizing the GA controller^39^. The merits of this controller are more efficient, highly robust, good dynamic response, and fast MPP tracking speed. However, this controller gives fluctuating load voltages under sudden variations in the water membrane content of the fuel stack.

The metaheuristic optimization techniques are used in the fuzzy logic system to improve the operating efficiency of the hybrid renewable energy system. The fuzzy membership functions and the converter duty cycle are selected by utilizing the firefly optimization controller, PSO, and teaching learning-based controller^45^. Here, all three optimization algorithms' comprehensive analysis has been done in terms of MPP tracking speed, oscillations across MPP, and convergence time of the controllers. From the simulation results analysis, the authors concluded that the teaching–learning optimization controller along with fuzzy gives the high accuracy of MPP tracking and very low steady-state oscillations across the MPP. However, this controller required multiple iterations to find out the suitable membership functions for the fuzzy logic controller. In^46^, the researchers studied the farmland fertility algorithm-based fuzzy controller for finding the suitable maximum power point of the renewable energy fed grid connected system under different operating temperatures, and irradiation conditions. Here, the farmland fertility algorithm tunes the fuzzy parameters effectively to improve the performance efficiency of the overall system under various atmospheric conditions. Also, this controller performance is compared with the other six conventional controllers in terms of operating efficiency, and tracking speed of MPP. Based on the comprehensive results, the working efficiency of the farmland fertility controller is 95%. Similarly, the other metaheuristic-optimized fuzzy logic controllers are explained in Table 1.

**Table 1:** Summary of metaheuristic optimized MPPT controllers.

References	Selected variables	MPPT category	MPPT	Type of converter	Major findings & limitations
Laxman et al.^40^	Current, & Voltage	Optimized Fuzzy with SHADE	Duty signal	Boost DC–DC Converter	As of now, most of the customers focusing on green building technology along with different renewable energy sources. In that, the fuel stack supplies continuous power to the customers to meet the peak load demand. Here, the successful history-dependent adaptive differential evolution methodology is used to optimize the fuzzy rules to obtain the suitable duty value for the power converter. The applications of this hybrid MPPT controller are battery charging and traffic signal monitoring
Yahiaoui et al.^41^	Current, & Voltage & Temperature	KHA with Fuzzy logic	Duty signal	Buck-Boost	Based on the environmental conditions, the solar and fuel cells supply power with different MPPs. As a result, the efficiency of the overall system is reduced. So, the krill herd-based fuzzy logic controller is interfaced with the three-phase grid-connected system for supplying the constant voltage to the load. Here, there are two sensors are used to sense the power and voltage for generating the suitable duty cycle to the converter
Selman et al.^42^	Current, & Voltage	Incremental conductance with Fuzzy	Duty signal	Quadratic Boost Converter	The incremental conductance-based fuzzy controller is used in the PV-based standalone system for reducing the MPP tracking time. The features of this controller are high MPP tracking speed, more accuracy, easy adaptability, and good dynamic response. Also, this hybrid controller reduces the voltage stress on the power semiconductors. Here, the quadratic converter is used for enhancing the voltage gain of the source, and it works effectively for low supply voltage. However, this converter needed a high installation cost, and the size is also very high
Priyadarshi et al.^43^	Current, & Voltage	Slider with fuzzy	Duty signal	Boost DC–DC Converter	In this work, the researchers utilized the ANFIS methodology to determine the peak power of the phosphoric acid fuel network. The disadvantages of ANFIS are more time for obtaining the uniform converter voltage, plus working under constant water membrane conditions of the fuel stack. So, the authors combined the ANFIS with a genetic controller to increase the overall system efficiency
Tao et al.^44^	Membrane water, Partial pressure	Whale Optimization Search	Duty signal	Buck-Boost	This work presents the demand for renewable power sources and their efficiency problems. The whale controller collects the signals from the fuel cell which are partial hydrogen pressure, water membrane, plus temperature to enhance the lifetime of the cell. Also, this controller provides a cost-effective solution for the nonlinear issue of the fuel stack

## Mathematical analysis of PEMFS

In the present EV industry, fuel stacks are more popular because of their merits such as uniform voltage production, easy fuel fill-up, better thermo dynamic response, and ease of handling. The Phosphoric acid-based fuel device is utilized for 100–400 kW commercial applications because of its more effective operation at medium power range and provides maximum efficiency at various water membrane values^47^. Also, it is used for residual systems, trains, buses, boats, automobiles, etc. However, this fuel stack has high volume density and is more costly because of the usage of platinum catalysts. The molten carbonate catalyst-based fuel technology is utilized in the steel industry network for producing power with low-level fluctuations. The features of this stack can produce electrical energy by using the different types of hydrocarbons, and natural gas. Also, it gives more power conversion efficiency by hybridizing the wind power networks. However, the life span of this stack is reduced when it works at high operating temperature values. Also, there is the possibility of a corrosion effect on the catalyst. To mitigate these problems, the solid oxide chemical composition-based fuel stack is developed in^48^ for off-the-grid remote power generation, military use, aeronautics, and space missions.

**Figure 2:**
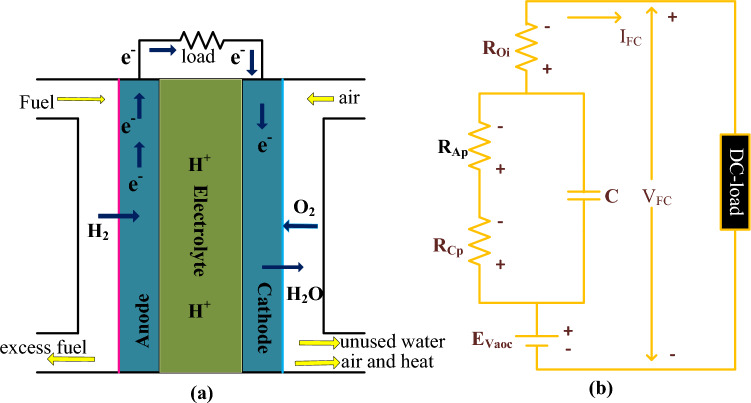
(**a**) Fuel stack device anode electrode and cathode operation and (**b**) Circuital indication polymer fuel device.

The solid oxide device features are high combined heat, more energy efficiency, long-term flexible response, more stable power generation, relatively produces very low hazardous gasses, and supplies energy as long as solid oxide is available^49^. However, the solid oxide device manufacturing cost is higher, and the rapid degradation of fuel stack performance. Also, this device needs more time to function. As a result, the entire system's compatibility, and mechanical issues occur. So, the polymer-based fuel device is selected in this article for analyzing the continuous different slope value-based cuckoo search methodology. The main features of this stack are easy sealing, required very thin polymer membrane, capable of working at high-level sub-freezing conditions, and quickly meeting the high energy demand application^50^. The functioning of this fuel device is illustrated in Fig. 2a, and its related circuital representation is mentioned in Fig. 2b. Here, the cathode, and anode electrodes are designed by using the platinum particles with the support of porous carbon. From Fig. 2a, the source side hydrogen (H_2_) gas is reacted with the natural oxygen (O_2_) which is coming from the cathode to produce the water (H_2_O). The developed fuel device model circuit is drawn in Fig. 2b. In Fig. 2, the terminologies R_Cp_, R_Ap_, and R_Oi_ are followed as concentrative region place resistor, active region place resistor, and ohmic place resistance, and their associated voltages are illustrated as E_Cp_, E_Ap_, and E_Oi_ respectively.

1 $${\text{H}}_{2} \to 2{\mathrm{H}}^{ + } + 2{\text{e}}^{ - }$$

2 $$2{\text{H}}^{ + } + 2{\mathrm{e}}^{ - } + 0.5{\text{O}}_{2} \to {\text{H}}_{2} {\mathrm{O}}$$

3 $${\mathrm{H}}_{2} + 0.5{\mathrm{O}}_{2} \to {\mathrm{H}}_{2} {\mathrm{O}} + {\mathrm{Energy}}$$

4 $${\mathrm{E}}_{{{\mathrm{total}}}} = {\mathrm{N }}\left( {{\mathrm{cells}}} \right){\mathrm{*V}}_{{{\mathrm{FC}}}}$$

5 $${\mathrm{V}}_{{{\mathrm{FC}}}} = {\mathrm{E}}_{{{\mathrm{Vaoc}}}} - {\mathrm{E}}_{{{\mathrm{Oi}}}} - {\mathrm{E}}_{{{\mathrm{Cp}}}} - {\mathrm{E}}_{{{\mathrm{Ap}}}}$$

6 $${\mathrm{E}}_{\mathrm{Vaoc}}=1.189-0.9{\mathrm{e}}^{-3}\left({\mathrm{T}}_{\mathrm{Fst}}-299.441\right)+4.281{\mathrm{e}}^{-5}\mathrm{Iog}({\mathrm{P}}_{{\mathrm{H}}_{2}}\sqrt{{\mathrm{P}}_{{\mathrm{O}}_{2}}})*{\mathrm{T}}_{\mathrm{Fst}}$$

7 $${\mathrm{P}}_{{{\mathrm{H}}_{2} }} = \frac{1}{2}{\mathrm{HumidityVapour}}_{{{\mathrm{Ad}}}}* {\mathrm{P}}_{{{\mathrm{H}}_{2} {\mathrm{O}}}}^{{{\mathrm{sat}}}} * \left( {\frac{1}{{\frac{{{\mathrm{HumidityV}}_{{\mathrm{A}}} {\mathrm{P}}_{{{\mathrm{H}}_{2} {\mathrm{O}}}}^{{{\mathrm{sat}}}} }}{{{\mathrm{P}}_{{\mathrm{A}}} }}\exp \left( {\frac{{1.644\left( {{\mathrm{I}}_{{{\mathrm{cell}}}} /{\mathrm{A}}} \right)}}{{{\mathrm{T}}_{{{\mathrm{Fst}}}} }}} \right)}}} \right)$$

8 $${\mathrm{P}}_{{{\mathrm{O}}_{2} }} = \frac{1}{2}{\mathrm{HumidityVapour}}_{{{\mathrm{Cd}}}} {\mathrm{*P}}_{{{\mathrm{H}}_{2} {\mathrm{O}}}}^{{{\mathrm{sat}}}} \left( {\frac{1}{{\frac{{{\mathrm{HumidityV}}_{{{\mathrm{Cd}}}} *{\mathrm{P}}_{{{\mathrm{H}}_{2} {\mathrm{O}}}}^{{{\mathrm{sat}}}} }}{{{\mathrm{P}}_{{{\mathrm{Cd}}}} }}\exp \left( {\frac{{4.092{*}\left( {{\mathrm{I}}_{{{\mathrm{cell}}}} /{\mathrm{A}}} \right)}}{{1.198{\mathrm{*T}}_{{{\mathrm{Fst}}}} }}} \right)}}} \right)$$

9 $${\mathrm{E}}_{\mathrm{AP}}={\mathrm{S}}_{1}+{\mathrm{S}}_{2}*{\mathrm{T}}_{\mathrm{Fst}}+({\mathrm{S}}_{3}+{\mathrm{S}}_{4}){*\mathrm{T}}_{\mathrm{Fst}}\mathrm{log}({\mathrm{C}}_{{\mathrm{O}}_{2}}+{\mathrm{I}}_{\mathrm{FC}})$$

10 $${\mathrm{E}}_{\mathrm{Cp}}=-\frac{\mathrm{R}*{\mathrm{T}}_{\text{Fst}}}{\text{N}(\mathrm{cells})*\mathrm{F}}\mathrm{Iog}\left(1-\frac{\mathrm{J}}{{\text{J}}_{\text{max}}}\right)$$

11 $${\text{E}}_{\text{Oi}}={\mathrm{I}}_{\mathrm{cell}}*({\mathrm{R}}_{\mathrm{aeff}}+{\text{R}}_{\text{ceff}})$$

12 $${\text{C}}_{{{\text{O}}_{2} }} = \frac{{{\mathrm{P}}_{{{\mathrm{O}}_{2} }} }}{{4.9987{\mathrm{e}}^{6} *\exp ( - 497.89/{\mathrm{T}}_{{{\mathrm{Fst}}}} )}}$$

13 $$\text{J}=\frac{{\text{I}}_{\text{cell}}}{\mathrm{A }(\mathrm{area})}$$

14 $${\mathrm{R}}_{\mathrm{ef}}=\frac{{\upgamma }_{\text{ef}}\mathrm{Q }(\mathrm{charge})}{\mathrm{A }(\text{area})}$$

15 $${\upgamma }_{{{\mathrm{ef}}}} = \frac{{180.98{ }\left[ {1 + 0.091{\mathrm{X}} + 0.489{*}({\mathrm{T}}_{{{\text{Fst}}}} /303)^{2} {\text{*J}}^{2.5} } \right]}}{{\left( {{\text{W}} - 0.651 - 3{\mathrm{J}}} \right)\exp \left( {\frac{{4.011\left( {{\mathrm{T}}_{{{\mathrm{Fst}}}} - 304} \right)}}{{{\mathrm{T}}_{{{\mathrm{Fst}}}} }}} \right)}}$$

**Figure 3:**
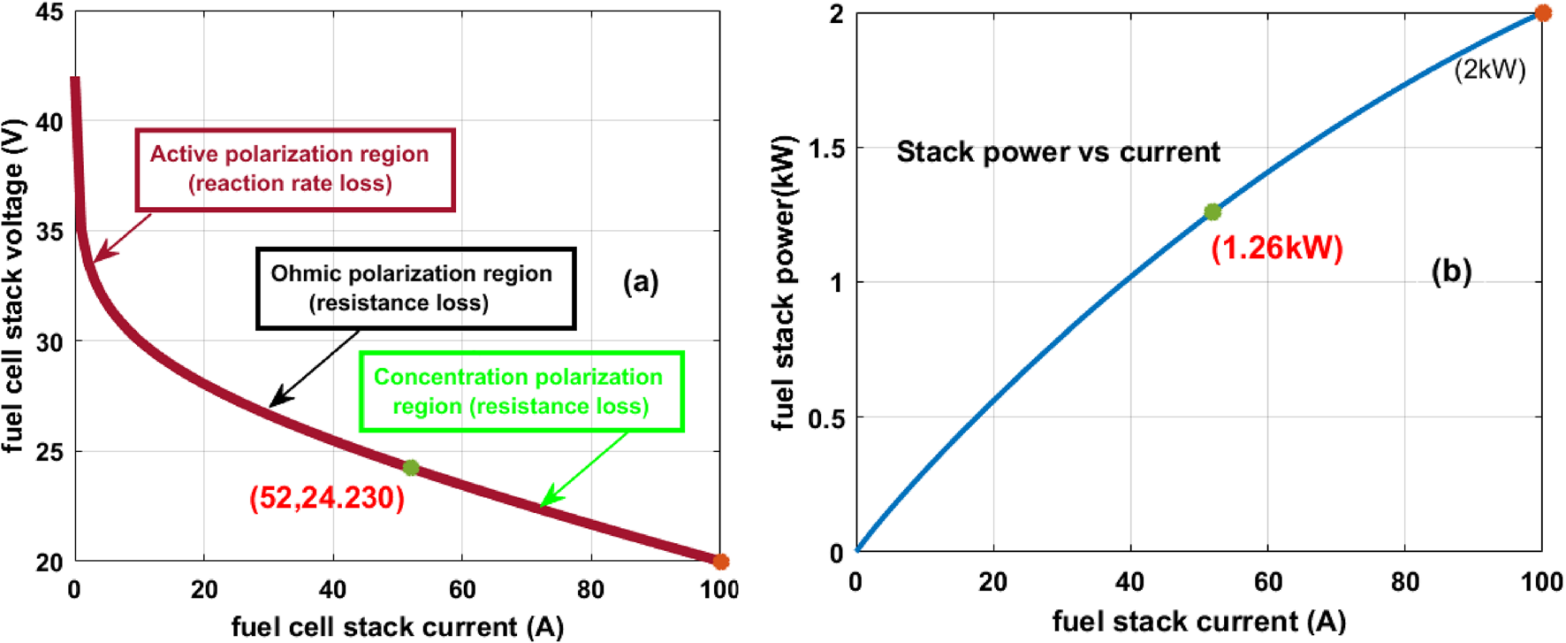
(**a**) I–V nonlinear curve of the Fuel stack. (**b**) I–P nonlinear curve of the Fuel stack.

From Eqs. (4), and (5), the variables “N”, E_Vaoc_, V_FC_, and T_Fst_ are defined as the overall cells selected for the development of the polymer membrane device, voltage of the cell at open-circuited, single cell voltage, and functioning temperature of the fuel stack. Similarly, the terminologies P_O2_, P_H2_, and E_total_ are named as pressure occurred due to the oxygen, hydrogen, and overall fuel stack voltage. Finally, I_cell_, J, A, Q, R_aeff_, R_ceff_, S_1_, S_2_, S_3_, S_4_ are illustrated as each fuel device current, current density, area of electrode, effective resistances of anode, cathode, and fuel stack empirical thermal coefficients. Inside the fuel stack gas constant (R), Faraday (F) constants are helpful for the thermodynamic analysis of the fuel stack. The selected fuel model design parameters are explained in Table 2, and its available power curves are represented in Fig. 3a, and b. From Fig. 3a, the maximum possible voltage (V_FMPP_), and current (I_FMPP_) of the fuel network are 24.230 V, and 52A respectively. The operating peak power (P_FMPP_) of the system is 1.26 kW.

**Table 2:** The fuel stack network utilized parameters for testing the MPPT controller.

Variables	Values
Identified fuel stack network power (P_FMPP_)	1.26 k Watts
Identified fuel stack network voltage (V_FMPP_)	24.23 Volts
Identified fuel stack network current (I_FMPP_)	52Ampere
Natural air rate of flow in the system (lpm)	2489
Maximum possible airflow value	4702
The overall system used fuel cells (N)	42
H_2_ flow in the fuel network at nominal condition	1.48 bar
O_2_ flow in the fuel network at nominal condition	1 bar
Inside the fuel stack, the available gas constant (R)	82.7992 [J mol^−1^ K^−1^]
Faraday constant selected for PEMFS (F)	97,022.877 [C mol^−1^]
Chemical oxidization percentage in the fuel network	20.97%
Overall chemical decomposition percentage	99.960%
In the fuel network, utilized hydrogen value	99.66%
In the fuel network, utilized oxygen value	59.3%
Available Nerst potential across the electrodes (V_FC_)	1.189 V

## Development of different power point tracking methods

All renewable power systems supply a fluctuating nonlinear voltage which is not used for any industry application. So, the renewable energy systems are designed by considering the MPPT technology for achieving the maximum possible voltage from the source^51^. Here, the fuel stack technology is working as a renewable energy source and its current production is optimized by considering the nature-inspired MPPT controllers which are discussed in Section “Literature review on power point tracking methods”. In this work, the continuous different slope value-based cuckoo search related power point identifier is developed for the nonlinear behavior-based fuel network system and it is investigated along with the Modified Differentiated Value based Perturb & Observe (MDV based P&O), Continuous Variable Step Value based Incremental Conductance (CVSV based IC), Modified Weight Adjustment based Radial Basis Functional Network (MWA based RBFN), Adaptive Continuous Step Change based Fuzzy Logic Controller (ACSC based FLC), and Self Step Adjustment based Particle Swarm Optimization (SSA based PSO). These algorithms are compared in terms of tracking time of the peak power point, convergence speed, maximum power extraction efficiency, plus dependency on the fuel stack implementation.

### Modified differentiated value-based perturb & observe controller

**Figure 4:**
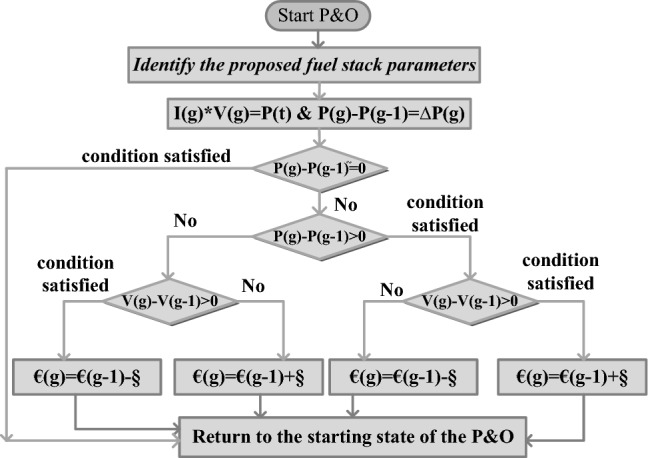
Identification of optimal converter duty value by using the P&O controller.

P&O is the very general MPPT concept that is utilized for peak power extraction from the renewable power network. This method is applicable only for the static fuel stack operating water membrane conditions. The advantages of this controller are quite simple structure, fewer element requirements, easy handling, and low cost for manufacturing. However, this controller faces the issue of more oscillations and convergence time. So, the modified step value-dependent P&O concept is introduced in^52^ for the wind/solar/fuel stack smart grid power network to obtain the optimal voltage of the triple-phase power converter circuit. Here, the fuel stack impedance continuous perturbation has been done to optimize the tracking speed of the power point tracking controller. This controller utilizes the variable step value and system parameters for reaching the peak power demand. Here, the converter duty signal is obtained by adjusting the equivalent consumer impedance as mentioned in Fig. 4. From Fig. 4, the terms §, and € are the step value, and updated duty signal of the converter. Finally, the converter generated previous and present voltages and powers are V(g), P(g), V(g − 1), plus P(g − 1).

16 $$\EUR\left( {\text{g}} \right) = \EUR\left( {{\mathrm{g}} - 1} \right) + {\rm{\S}} \left( {\frac{{{\mathrm{P}}\left( {\mathrm{g}} \right) - {\text{P}}\left( {{\text{g}} - 1} \right)}}{{{\text{V}}\left( {\mathrm{g}} \right) - {\mathrm{V}}\left( {{\mathrm{g}} - 1} \right)}}} \right)$$

17 $$\EUR\left( {\mathrm{g}} \right) = \delta \left( {{\text{g}} - 1} \right) - {\rm{\S}} \left( {\frac{{{\text{P}}\left( {\mathrm{g}} \right) - {\mathrm{P}}\left( {{\mathrm{g}} - 1} \right)}}{{{\mathrm{V}}\left( {\text{g}} \right) - {\text{V}}\left( {{\mathrm{g}} - 1} \right)}}} \right)$$

### Continuous variable step value-based incremental conductance

**Figure 5:**
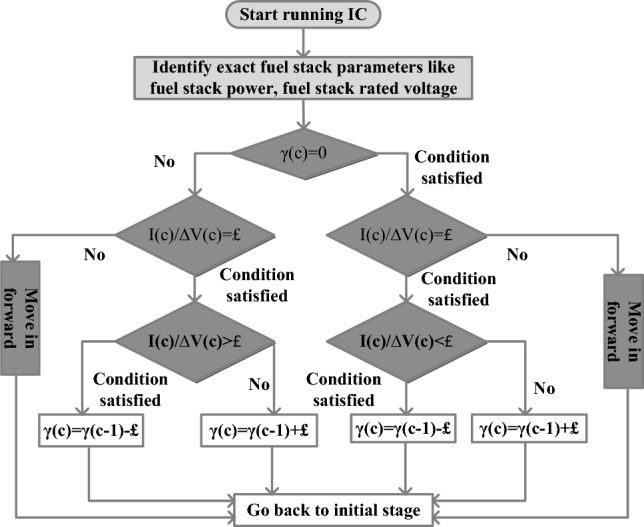
Identification of the fuel stack functioning point by using the CVSV-IC.

The P&O concept provides more fluctuated power and takes more time for convergence. So, the continuous variable step value-based incremental conductance methodology is integrated with the smart grid power supply network for tunning the functioning point of the hybrid fuel stack energy production network thereby running the automotive network with more efficiency. This type of methodology is applied for the sunlight traffic controlling network, and water pumping networks. In^53^, the CVSV-IC block is placed near the fuel cell-based battery charging station for controlling the vehicle voltage rating. This controller purely monitors the I–V curve inductance. The present evaluated I–V curve conductance is positive high then the IC method moves in forward functioning state. Suppose, the determined fuel stack I–V curve conductance is negative low then IC starts functioning in the opposite direction. The identification of the working duty signal of the power conventional converter is illustrated in Fig. 5. The change of instant conductance-based duty (ϒ) value of the converter is mentioned in Eq. (18). Based on Eq. (19), the terminologies ϒ(c − 1), P(c − 1), and V(c − 1) are the previous stored duty value, power, and stored fuel stack voltage. Finally, the variable ${\pounds}$ defines the step conductance of the fuel stack.

18 $$\Upsilon \left( {\mathrm{c}} \right) = \Upsilon \left( {{\mathrm{c}} - 1} \right) + {{\pounds*}}\left( {\frac{{{\mathrm{P}}\left( {\mathrm{c}} \right) - {\mathrm{P}}\left( {{\text{c}} - 1} \right)}}{{{\text{V}}\left( {\text{c}} \right) - {\text{V}}\left( {{\mathrm{c}} - 1} \right)}}} \right)$$

19 $$\Upsilon \left( {\mathrm{c}} \right) = \Upsilon \left( {{\mathrm{c}} - 1} \right) - {{\pounds*}}\left( {\frac{{{\mathrm{P}}\left( {\mathrm{c}} \right) - {\mathrm{P}}\left( {{\mathrm{c}} - 1} \right)}}{{{\mathrm{V}}\left( {\text{c}} \right) - {\text{V}}\left( {{\text{c}} - 1} \right)}}} \right)$$

### Modified weight adjustment based radial basis functional network

**Figure 6:**
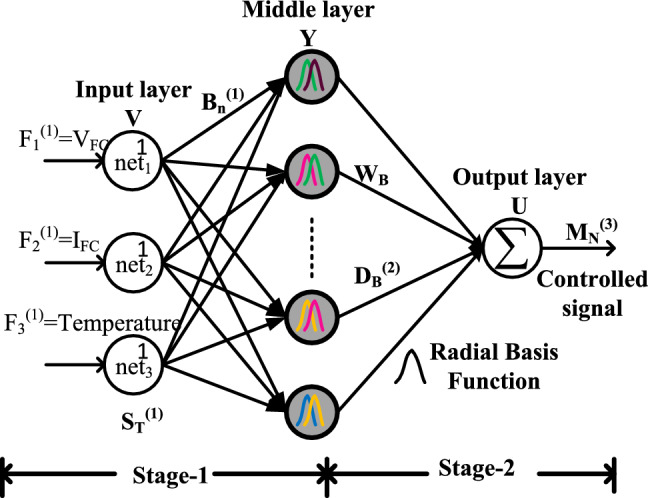
Modified weight adjustment based RBFN MPPT controller.

The neural networks are developed based on the human brain's functioning nature. All the neural controllers consist of multiple neurons which are correlated with each and another neuron. These neurons start working by exchanging their searching data. The neural controller tries to obtain the required object by selecting the various activation functions. In^54^, the authors referred to the multiple layers neural network which consists of 627 neurons and it collects the fuel stack power, oxygen decomposition rate, and hydrogen decomposition rates for supplying the suitable duty signal to the quadratic converter. This network needs a huge amount of training data, takes a greater number of iterations, has less convergence speed, and has low accuracy. So, the MWA-RBFN methodology proposed in^55^ for EV battery charging. This controller has three layers which are identified as the source layer, radial basis function-based hidden layer, and output layer. Here, only two fuel stack constraints are utilized as given in Fig. 6. This MWA-RBFN works efficiently at rapid variation of water membrane content, and different hydrogen decomposition rates.

20 $${\text{net}}_{\mathrm{V}}^{(1)}={\mathrm{F}}_{\mathrm{V}}^{1}(\mathrm{i}); \quad \mathrm{i}=1,2,3,4,...,\mathrm{k}$$

21 $${\mathrm{D}}_{\mathrm{n}}^{\left(1\right)}\left(\mathrm{i}\right)={\mathrm{f}}_{\mathrm{V}}^{\left(1\right)}\left({\mathrm{net}}_{\mathrm{V}}^{\left(1\right)}\left(\mathrm{i}\right)\right)={\mathrm{net}}_{\mathrm{V}}^{1}\left(\mathrm{i}\right);\quad \mathrm{ V}=1,2,3$$

22 $${\mathrm{net}}_{\mathrm{B}}^{(2)}(\mathrm{i})=-(\mathrm{F}-{\upmu }_{\mathrm{B}}{)}^{\text{T}}*{\sum }_{\mathrm{B}}(\mathrm{F}-{\upmu }_{\mathrm{B}})$$

23 $${\mathrm{D}}_{\mathrm{B}}^{\left(2\right)}(\mathrm{i})={\mathrm{f}}_{\mathrm{B}}^{\left(2\right)}({\mathrm{net}}_{\mathrm{B}}^{\left(2\right)}(\mathrm{i}));\quad \mathrm{B}=1,2,3,4,\ldots,9$$

24 $${\upmu }_{{\mathrm{B}}} = \left[ {\begin{array}{*{20}c} {{\upmu }_{{1{\mathrm{B}}}} } & {{\upmu }_{{2{\mathrm{B}}}} } & {{\upmu }_{{3{\mathrm{B}}}} } & \ldots & {{\upmu }_{{{\mathrm{VB}}}} } \\ \end{array} } \right]$$

25 $$\mathop \sum \limits_{{\mathrm{B}}} = {\mathrm{diag}}\left[ {\begin{array}{*{20}c} {{\raise0.7ex\hbox{$1$} \!\mathord{\left/ {\vphantom {1 {\eta_{{1{\mathrm{B}}}}^{2} }}}\right.\kern-0pt} \!\lower0.7ex\hbox{${\eta_{{1{\mathrm{B}}}}^{2} }$}}} & {{\raise0.7ex\hbox{$1$} \!\mathord{\left/ {\vphantom {1 {\eta_{{2{\text{B}}}}^{2} }}}\right.\kern-0pt} \!\lower0.7ex\hbox{${\eta_{{2{\text{B}}}}^{2} }$}}} & {{\raise0.7ex\hbox{$1$} \!\mathord{\left/ {\vphantom {1 {\eta_{{3{\text{B}}}}^{2} }}}\right.\kern-0pt} \!\lower0.7ex\hbox{${\eta_{{3{\mathrm{B}}}}^{2} }$}}} & \ldots & {{\raise0.7ex\hbox{$1$} \!\mathord{\left/ {\vphantom {1 {\sigma_{{{\mathrm{ND}}}}^{2} }}}\right.\kern-0pt} \!\lower0.7ex\hbox{${\sigma_{{{\mathrm{ND}}}}^{2} }$}}} \\ \end{array} } \right]$$

26 $${\mathrm{net}}_{\mathrm{N}}^{(3)}={\sum }_{\mathrm{k}}{\mathrm{B}}_{\mathrm{k}}{\mathrm{D}}_{\mathrm{k}}^{2}(\mathrm{i});\quad \mathrm{i}=1,2,3,4,5,\ldots,\mathrm{k}$$

27 $${\mathrm{O}}_{\mathrm{U}}^{(3)}(\mathrm{i})={\mathrm{f}}_{\mathrm{U}}^{(3)}(({\mathrm{net}}_{\mathrm{U}}^{3}(\mathrm{i}))={\mathrm{net}}_{\mathrm{U}}^{3}(\mathrm{i})$$

where the terms V, Y, U, and O are the names of the layer, and B, N, and D are the weights. The variables ${\mathrm{F}}_{\mathrm{V}}^{1}\left(\mathrm{i}\right),$ ${\mathrm{net}}_{\mathrm{V}}^{\left(1\right)}\left(\mathrm{i}\right)$ are the net values of the supply layer.

### Adaptive continuous step change-based fuzzy logic controller

**Figure 7:**
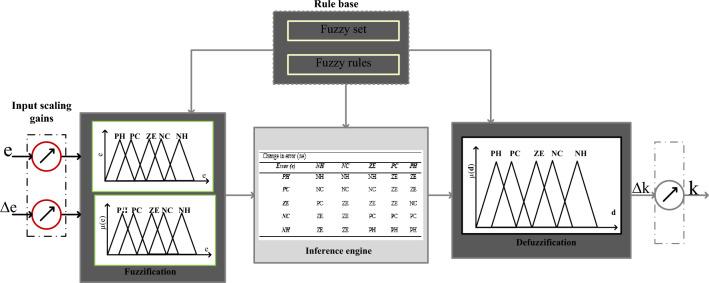
Adaptive continuous step change-based fuzzy logic controller.

From the literature study, the neural controller complexity rises by improving the total number of input signals. Also, this network layer rises by selecting a greater number of neurons. In addition, the network needs more data to obtain the required switching pulses for the interleaved converter^56^. The major issues of neural methods are overfitting issues, and improper membership function selection creates the system voltage oscillations. Also, the neural networks work for the fixed input and output signals. So, the limitations of neural networks are compensated by selecting the fuzzy logic technology. The adaptive continuous step changed fuzzy block is connected to the grid-interfaced water pumping network for handling the uncertainty of the hybrid power production network. Fuzzy logic is developed based on the mathematical formulas and its membership functions are selected based on the application necessity. In addition, fuzzy is a very powerful tool for finding the optimal solution for three-dimensional issues. The fuzzy application for the fuel stack connected hybrid power supply network is discussed in Fig. 7. From Fig. 7, the fuzzification system transfers the real signals to the linguistic signals and the inference system utilizes the if–then concept for finding the suitable membership values. Finally, the defuzzification system helps to transfer the linguistic values into real constants.

### Self step adjustment based particle swarm optimization controller

**Figure 8:**
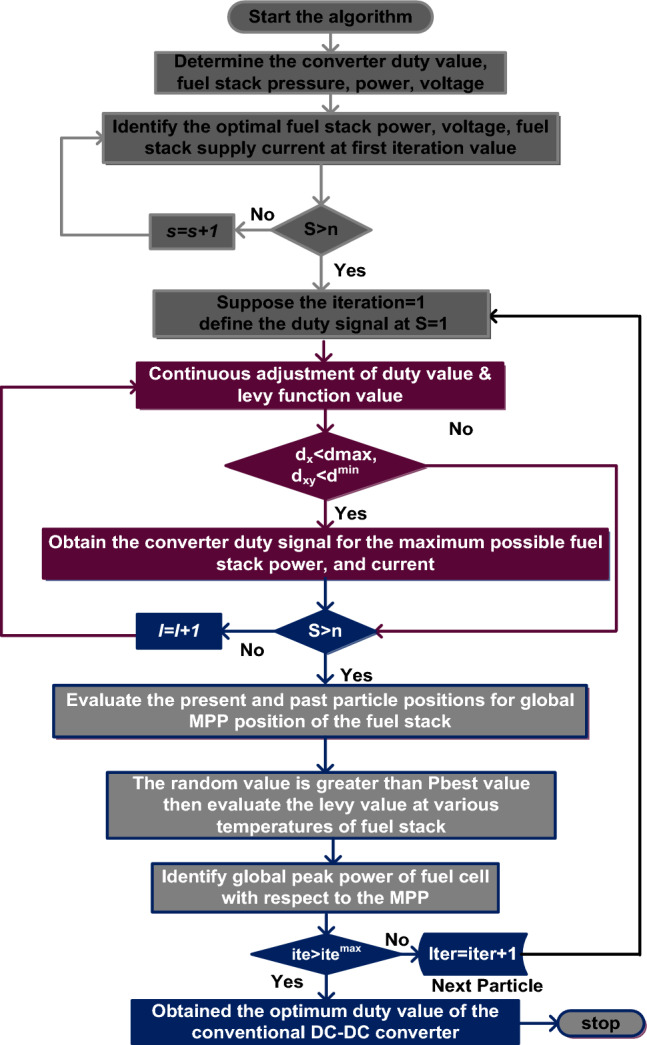
Self-step adjustment-based particle swarm optimization controller.

The neural, and fuzzy networks need high expertise candidates, and the selection of fuzzy membership function is the major problem in the fuzzy MPPT block. So, the SSA-PSO technology is developed in the fuel/solar/wind power network for sorting out the nonlinear problem of the overall system. This method selects the all-fuel stack-related constraints which are pressure constant, hydrogen chemical rate, pressure of oxygen, fuel stack deliver voltage, and fuel stack current for finding the accurate MPP place of the system^57–59^. In this algorithm, at the initial state, all the particles run at different distances, and various velocities from the required object point. In the 2^nd^ iteration, the particles share their search data regarding the location of global MPP. After a few numbers of iterations, the PSO methodology tries to move to the actual power point position. The particle velocity in the PSO is adjusted by utilizing Eq. (28) and its change of distance is illustrated in Fig. 8.

28 $${\mathrm{S}}^{\mathrm{z}+1}=\mathrm{W}*{\mathrm{S}}_{\mathrm{z}}^{i}+{\mathrm{H}}_{1}{\mathrm{K}}_{1}\left({\mathrm{P}}_{\mathrm{b}\_\mathrm{i}}-{\mathrm{n}}_{\mathrm{i}}^{\mathrm{z}}\right)+{\mathrm{H}}_{2}{\mathrm{K}}_{2}\left({\mathrm{G}}_{\mathrm{b}\_\mathrm{i}}-{\mathrm{n}}_{\mathrm{i}}^{\mathrm{z}}\right)$$

29 $${\mathrm{n}}^{\mathrm{z}+1}={\mathrm{Y}}_{\mathrm{i}}^{\mathrm{z}}+{\mathrm{S}}_{\mathrm{i}}^{\mathrm{z}+1}$$

where, the terms S^z+1^, and n^z+1^ are the agents' running velocity and distance at iteration “z”. Similarly, the local power and global power of the fuel stack at the iteration ‘z’ are defined as P_b_i,_ plus G_b_i_. Finally, the H_1_, H_2_, K_1_, and K_2_ are the PSO accelerated values and random initiated values.

### Continuous different slope value-based cuckoo search related power point identifier

**Figure 9:**
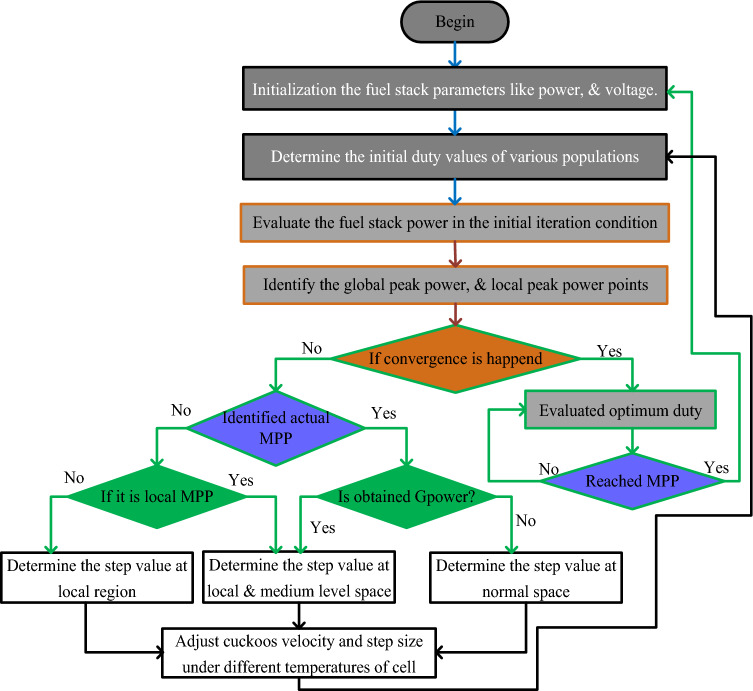
Continuous different slope value-based cuckoo search related power point identifier.

The disadvantage of the PSO method is there is the possibility of settling the MPP point at any local place of the I-V curve of the fuel stack system, and has a low convergence rate for high dimensional search space. So, the CDSV with CSM is utilized in the proposed fuel stack power system for obtaining the possible peak power from the system. In this search concept, the selected cuckoos should have more quality eggs before the algorithm moves to another stage. In the second state, one cuckoo should give only one possible peak solution. In the final stage of the cuckoo search, the levy concept is applied to get the various step values in the searching mechanism. In this algorithm, the system current, and fuel stack voltages are selected for obtaining the suitable duty value to the conventional converter. The working stages of the cuckoo search technique are illustrated in Fig. 9.

30 $${\mathrm{Levy }}\left( \Upsilon \right) = {\mathrm{Length}}^{ - \Upsilon } ;\;\;1.5 < \Upsilon < 3.5$$

31 $${\mathrm{M}}_{{\mathrm{n}}}^{{{\mathrm{n}} + 1}} = {\mathrm{M}}_{{\mathrm{n}}}^{{\mathrm{n}}} + \alpha \oplus {\mathrm{levy}}\left( \Upsilon \right)$$

32 $${\mathrm{l}} = \alpha_{0} \left( {{\mathrm{q}}_{{{\mathrm{best}}}} - {\mathrm{q}}_{{\mathrm{n}}} } \right) \oplus {\mathrm{levy}}\left( \Upsilon \right) \approx {\mathrm{c}}\left( {\frac{{\mathrm{u}}}{{v^{{{\raise0.7ex\hbox{$1$} \!\mathord{\left/ {\vphantom {1 {\rm{\S}} }}\right.\kern-0pt} \!\lower0.7ex\hbox{$ {\rm{\S}} $}}}} }}} \right)*\left( {{\mathrm{q}}_{{{\mathrm{best}}}} - {\mathrm{q}}_{{\mathrm{n}}} } \right)$$

33 $$\Psi = {\mathrm{a}}\left( {0,{ }\lambda_{{\mathrm{u}}}^{2} } \right),\;\;\;{\upomega } = {\mathrm{b}}\left( {0,{ }\lambda_{{\mathrm{v}}}^{2} } \right)$$

From Eq. (30), the terminologies $\Upsilon$, j, M, Ψ, ʎ, and ω are defended as the levy value, iteration value, length of cuckoo, curve function, and distribution waveforms. Finally, the parameters u, v, q, and n are the distribution curve constants, global power of the fuel network, and utilized cuckoo number.

## Development of Selected Power DC–DC Converter Circuit

**Figure 10:**
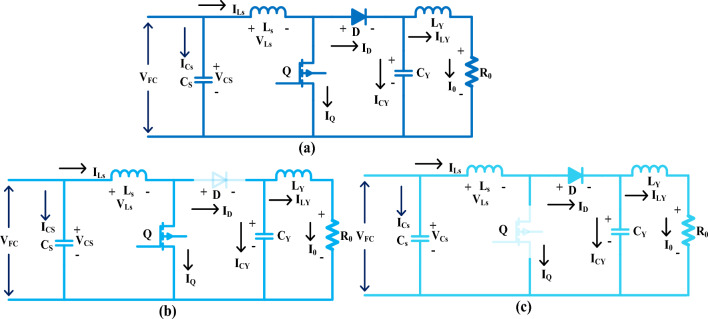
(**a**) Utilized single switch circuit, (**b**) functioning state, and (**c**) blocking mode.

As of the literature study, the isolated circuits have the problems of more weight, more power electronics switches utilization, excessive installation size, and must and should require one additional transformer^60–63^. Due to these many powers’ devices utilization, the isolated circuits take more power to start functioning. As a result, the entire power production network's functioning efficiency is low. So, this methodology is not referred to in automotive vehicles. Here, the single switch-oriented power converter is applied to the fuel stack network to optimize the power flow of the load which is discussed in Fig. 10a. This converter enhances load voltage and reduces the fuel network current. The merits of this technology are easy to model, flexible operation, one driver circuit is enough to run the switch and high robustness. The cuckoo search technology is selected to turn on the switch as given in Fig. 10b, and the driver circuit stops giving the pulses to the converter then the converter moves in an ideal state. The voltage conversion value of the DC–DC converter circuit is mentioned in Eq. (35).

34 $${\mathrm{DT}}_{\mathrm{m}}*{\mathrm{V}}_{\mathrm{FC}}+\left(1-\mathrm{D}\right){\mathrm{T}}_{\mathrm{m}}*\left({\mathrm{V}}_{\mathrm{FC}}-{\mathrm{V}}_{0}\right)=0$$

35 $$-{\mathrm{I}}_{0}\mathrm{D}*{\mathrm{T}}_{\mathrm{m}}+(\left({\mathrm{I}}_{\mathrm{FC}}-{\mathrm{I}}_{0}\right)*\left(1-\mathrm{D}\right){\mathrm{T}}_{\mathrm{m}}=0$$

36 $${\mathrm{V}}_{0} = {\raise0.7ex\hbox{${{\mathrm{V}}_{{{\mathrm{FC}}}} }$} \!\mathord{\left/ {\vphantom {{{\mathrm{V}}_{{{\mathrm{FC}}}} } {\left( {1 - {\mathrm{D}}} \right)}}}\right.\kern-0pt} \!\lower0.7ex\hbox{${\left( {1 - {\mathrm{D}}} \right)}$}}{ }\& ,\;{\mathrm{ I}}_{0} = {\mathrm{I}}_{{{\mathrm{FC}}}} \left( {1 - {\mathrm{D}}} \right)$$

where the variables L_s_, C_s_, Q, D, C_v_, and L_v_ are named as source inductor, input capacitor, switch, diode, load capacitor, and load inductor. Finally, the T_m_ and D are the selected time and converter operating duty values.

## Discussion of proposed system results

The single switch power DC–DC circuit is designed along with the fuel stack network by using the Simulink software. The converter parameter C_S_ is selected as 125 μF which is applied to the fuel network topology for maintaining the uniform supply voltage. Also, it helps to protect the MOSFET device from the quick changes of the grid voltages. Here, the Metal Oxide Semiconductor Field Effect Transistor (MOSFET) is selected because of its merits are low conducting resistance, low sensitivity feature concerning the temperature, more source impedance, and works for high power rating applications. The supply inductor L_S_ value is 200 mH and this suppresses the variations of the fuel stack network currents. Also, it limits the sudden variation of the fuel stack currents. So, the diode (D) starts to move in an active conduction place. The load capacitor C_Y_, and load inductor L_Y_ values are 100 μF, plus 80 mH. Finally, the resistor value used for the study of continuous different slope value-based cuckoo search method is 48 Ω. The considered fuel network power rating is 1.26 kW, and it produces the maximum possible current, and fuel stack voltages are 52A plus 24.2302 V. The major advantages of this system are more power efficiency, compact size, plus good dynamic performance. However, in this work, the suggested system is studied at 275 K, 275 K, 305 K, and 335 K temperature values.

### Investigation of proposed CDSV with CSM at 275 K temperature

**Figure 11:**
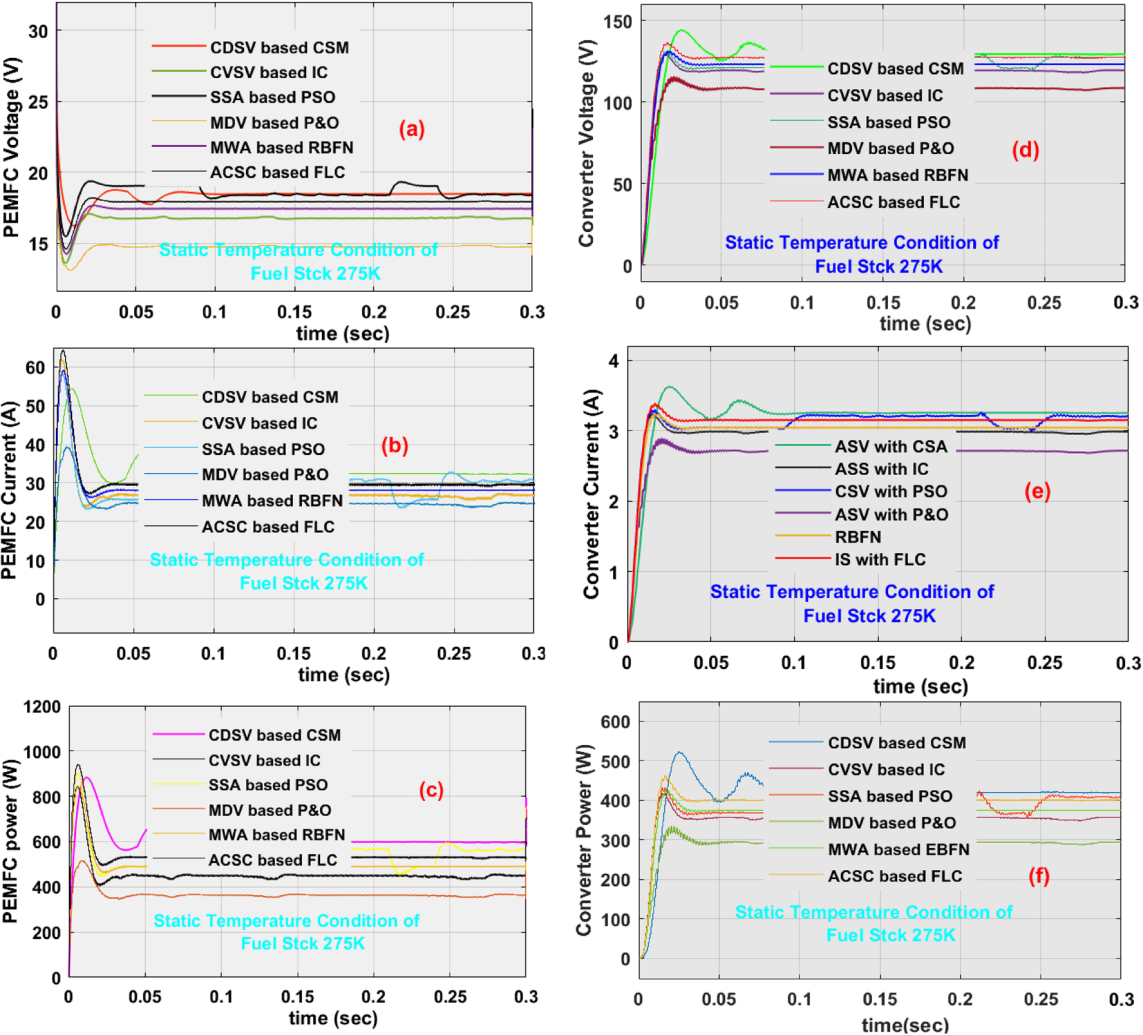
(**a**) Fuel power network voltage, (**b**) fuel power network current, (**c**) fuel power network power, (**d**) converter voltage, (**e**) converter current, and (**f**) power of converter at 275 K temperature.

**Figure 12:**
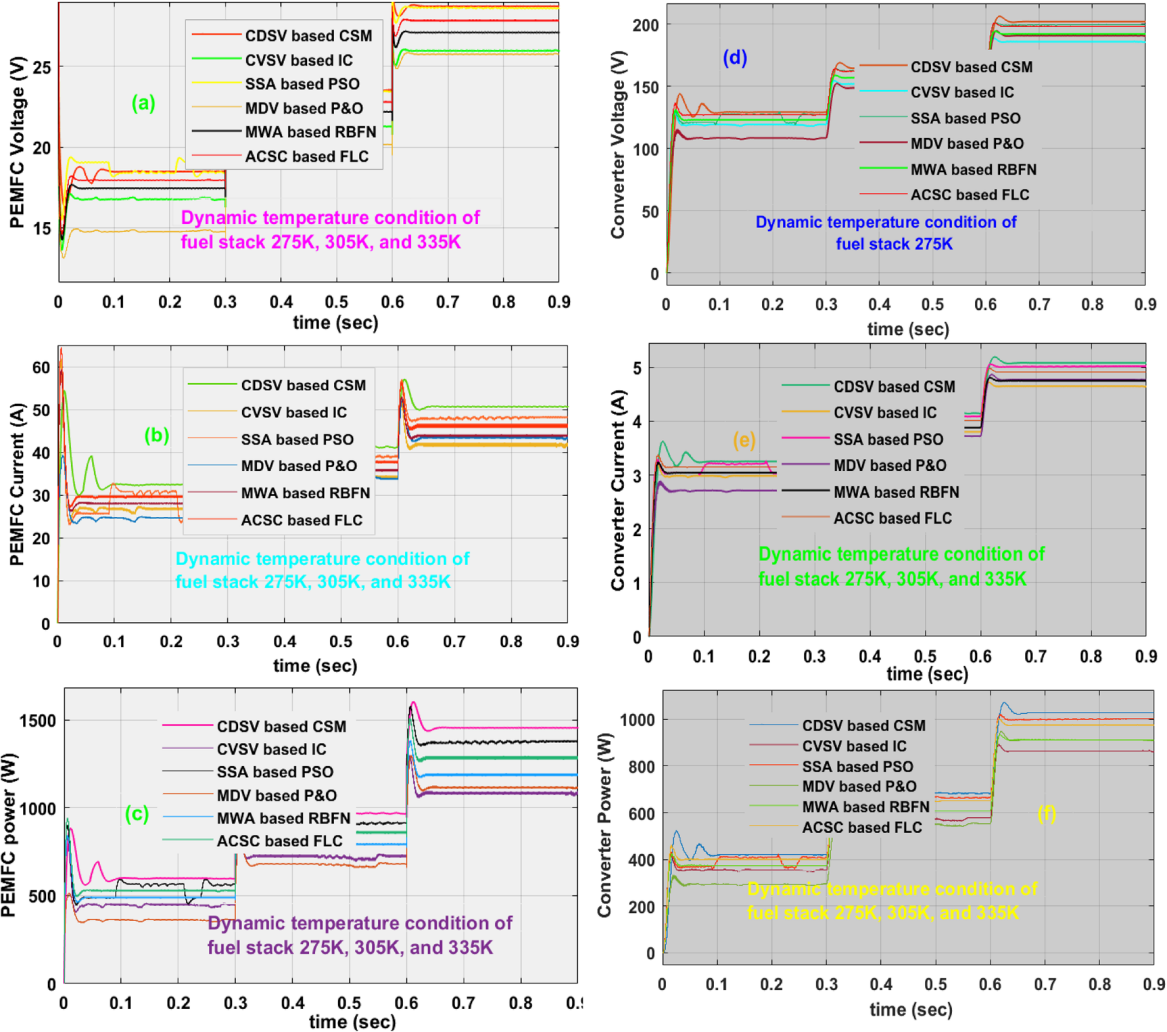
(**a**) Fuel power network voltage, (**b**) fuel power network current, (**c**) fuel power network power, (**d**) converter voltage, (**e**) converter current, and (**f**) power of converter at the quick change of temperature conditions.

Here, at the initial state of the proposed system, the MDV-based P&O, CVSV-based IC, MWA-RBFN, ACSC-based FLC, SSA-based PSO, and CDSV with CSM MPPT controllers identify the functioning point of the fuel stack with the tracking speed, and convergence efficiencies are 0.1231 s, 96.087%, 0.1127 s, 96.990%, 0.1124 s, 97.109%, 0.1207 s, 97.521%, 0.1120 s, 98.002%, 0.1081 s, plus 98.1056% respectively. Similarly, the power DC–DC circuits and fuel stack network obtained powers, voltages, and currents by selecting the MDV based P&O, CVSV based IC, MWA-RBFN, ACSC based FLC, SSA based PSO, and CDSV with CSM MPPT controllers are 395.07W, 124.07 V, 3.184A, 411.18W, 14.04 V, 29.27A, 399.14W, 124.77 V, 3.199A, 411.52W, 13.85 V, 29.71A, 410.55W, 125.55 V, 3.27A, 422.81W, 14.14 V, 29.89A, 418.99W, 127.10 V, 3.297A, 427.622W, 14.12 V, 30.27A, 420.14W, 126.88 V, 3.3112A, 428.27W, 14.134 V, and 30.33A respectively. The analysis of the entire fuel stack network at uniform working temperature value is illustrated in Table 3. From Table 3, the functioning point of the fuel network oscillates heavily by applying the MDV-based P&O, and CVSV-based IC. Also, these controllers are not useful for rapid changes in the fuel network operating thermal temperature coefficients. The implementation of the cuckoo search concept is very moderate. However, it provides very low distorted supply voltage waveforms. So, from the above simulative values, the proposed CDSV-based CSM is better suitable for the maximum possible power extraction from renewable energy networks. The fuel and DC–DC converter circuit power network voltages, currents, power waveforms are illustrated in Fig. 11a–f. Finally, from Fig. 12f, the ACSC-based FLC provides superior performance when associated with the MDV-based P&O, and CVSV-based IC controllers.

**Table 3:** Overall continuous different slope value-based cuckoo search method for fuel stack system.

MPPT	Fuel current	Fuel voltage	Fuel power	DC–DC current	DC–DC voltage	DC–DC Power	MPPT efficiency	Tracking speed	MPP Oscillations	MPPT design complexity
Overall fuel power system running temperature condition 275 K										
MDV based P&O	29.27 A	14.04 V	411.18 W	3.184 A	124.07 V	395.07 W	96.087%	0.1231 s	Excessive	Low level
CVSV based IC	29.71 A	13.85 V	411.52 W	3.199 A	124.77 V	399.14 W	96.990%	0.1127 s	Excessive	Low level
MWA-RBFN	29.89 A	14.14 V	422.81 W	3.270 A	125.55 V	410.55 W	97.109%	0.1124 s	Moderate	Moderate
ACSC based FLC	30.04 A	14.30 V	429.64 W	3.281 A	127.70 V	418.99 W	97.521%	0.1207 s	Moderate	Moderate
SSA based PSO	30.27 A	14.12 V	427.622 W	3.297 A	127.10 V	419.07 W	98.002%	0.1120 s	Moderate	Moderate
CDSV-CSM	30.33 A	14.134 V	428.27 W	3.3112 A	126.88 V	420.14 W	98.1056%	0.1081 s	Low level	Moderate
Overall fuel power system running temperature condition 305 K										
MDV based P&O	36.18 A	17.719 V	641.09 W	3.921 A	157.87 V	619.04 W	96.5621%	0.1230 s	Excessive	Low level
CVSV based IC	36.42 A	17.57 V	640.26 W	3.980 A	156.25 V	621.89 W	97.1321%	0.1218 s	Excessive	Low level
MWA-RBFN	37.52 A	17.06 V	640.3 W	3.997 A	155.90 V	623.14 W	97.324%	0.1217 s	Moderate	Moderate
ACSC based FLC	37.57 A	16.97 V	637.9 W	4.003 A	154.97 V	625.02 W	97.981%	0.118 s	Moderate	Moderate
SSA based PSO	39.12 A	16.34 V	639.61 W	4.107 A	152.95 V	628.17 W	98.211%	0.1101 s	Moderate	Moderate
CDSV-CSM	40.72 A	15.85 V	641 W	4.114 A	153.11 V	629.92 W	98.2706%	0.085 s	Low level	Moderate
Overall fuel power system running temperature condition 335 K										
MDV based P&O	43.99 A	21.25 V	934.93 W	4.803 A	189.59 V	910.63 W	97.421%	0.1229 s	Excessive	Low level
CVSV based IC	44.12 A	21.09 V	930.85 W	4.827 A	189.02 V	912.42 W	98.022%	0.1214 s	Excessive	Low level
MWA-RBFN	46.41 A	20.17 V	936.47 W	4.898 A	187.77 V	919.71 W	98.213%	0.1212 s	Moderate	Moderate
ACSC based FLC	46.48 A	19.96 V	928.10 W	4.971 A	185.71 V	923.19 W	99.4762%	0.114 s	Moderate	Moderate
SSA based PSO	46.50 A	20.37 V	947.26 W	4.982 A	189.22 V	942.72 W	99.521%	0.098 s	Moderate	Moderate
CDSV-CSM	46.52 A	20.66 V	961.47 W	5.012 A	191.83 V	958.21 W	99.786%	0.0826 s	Low level	Moderate

### Investigation of proposed CDSV with CSM at 275 K, 305 K, and 335 K

The identification of the exact functioning point of the fuel stack system under dynamic operating temperature values is a very challenging concept. The swarm and artificial intelligence concepts are used in this work for running the proposed power network at uniform output voltage. At 305 K, the obtained fuel stack and converters deliver currents, supply voltages, and powers by integrating the MDV based P&O, CVSV based IC, MWA-RBFN, ACSC based FLC, SSA-based PSO, and CDSV with CSM based power point identifiers are 36.18A, 17.719 V, 641.09W, 3.921A, 157.87 V, 619.04W, 36.42A, 17.57 V, 640.26W, 3.980A, 156.25 V, 621.89W, 37.52A, 17.06 V, 640.3W, 3.997A, 155.90 V, 623.14W, 37.57A, 16.97 V, 637.9W, 4.003A, 154.97 V, 625.02W, 39.12A, 16.34 V, 639.61W, 4.107A, 152.95 V, 628.17W, 40.72A, 15.85 V, 641W, 4.114A, 153.11 V, and 629.92W. Here, the MPPT tracking efficiency, and its related tracking speeds by selecting the MDV-based P&O, CVSV-based IC, MWA-RBFN, ACSC-based FLC, SSA-based PSO, and CDSV with CSM technologies are 96.5621%, 0.1230 s, 97.1321%, 0.12198 s, 97.324%, 0.1217 s, 97.981%, 0.118 s, 98.211%, 0.1101 s, 98.2706%, and 0.085 s respectively. The determined fuel network and power conversion circuits voltages, currents, and power waveforms are illustrated in Fig. 12a–f. Finally, the overall network investigation has been done at 335 K as mentioned in Table 3.

## Comprehensive investigation of MPPT controllers

### Tracking time of the fuel stack network MPP

From the results included in Table 3, the fuel network functioning point tracking times by suggesting the MDV-based P&O, CVSV-based IC, MWA-RBFN, ACSC-based FLC, SSA based PSO, and CDSV with CSM based power point identifiers under 335 K running temperature are 0.1229 s, 0.1214 s, 0.1212 s, 0.114 s, 0.098 s, and 0.0826 s respectively. So, the proposed Continuous Different Slope Value-based Cuckoo Search Method takes very little time to identify the exact MPP location of the utilized system.

### Oscillations of the fuel stack MPP position

The above simulation results proved that the modified differentiated value-based perturb & observe, and continuous variable step value-based incremental conductance controllers create more distortions in the system voltage because of its inaccurate power point identification when associated with the modified weight adjustment based radial basis functional network, adaptive continuous step change based fuzzy logic controller. Also, artificial intelligence-dependent methodologies need highly qualified persons to identify the proper dataset. The modified PSO technology can track the accurate MPP position by taking more number iterations. Due to this, the PSO convergence time increases. All these issues of the fuel stack network are limited by integrating the proposed cuckoo search methodology.

### MPPT dependency on the fuel stack network

The power point identifiers development completely depends on the category of fuel stack utilized, and its utilized chemical composition. The P&O controller works based on the fuel stack functioning voltages, and fuel stack powers, and it does not depend on the oxidization rate and utilized hydrogen decomposition. The neural networks and fuzzy methodologies start functioning based on the supply network parameters which are water membrane content value, working temperature of the fuel stack, supplying hydrogen pressure, and chemical reaction rate. So, these fundamental and artificial intelligence algorithms provide moderate efficiency when associated with nature-inspired algorithms. Also, the fuzzy network needs a highly knowledgeable person to identify suitable membership functions. If the utilized membership values are not proper then the fuzzy MPPT tracks the local MPP place instead of the required global MPP.

## Conclusion

The selected polymer membrane-based fuel stack fed power converter circuit along with the continuous different slope value-based cuckoo search method is analyzed by utilizing the Simulink window. In the first objective, the polymer membrane fuel cell technology is selected because of its advantages are high voltage density, low starting time, compact design and development, plus more efficiency for all temperature conditions. However, its power production is nonlinear fashion. So, the CDSV-based CSM is developed with the second objective of obtaining the maximum possible output power from the fuel system network. The merits of this proposed cuckoo search MPPT algorithm are high MPP identifying speed, more convergence rate, ability to function at any temperature conditions, best suitable for the dynamic functioning temperature conditions of the fuel stack, and providing the suitable duty signal to the converter. However, this algorithm development complexity is moderate when associated with the fundamental controllers. Finally, the main issue of the fuel stack is more amount of production current which is not acceptable for the local consumers. So, the converter technology is placed near the fuel stack network to enhance the supply voltage profile and optimize the load current.

## Future scope of the article

The present investigated nature-inspired algorithm-based power point identifiers development cost is more, and it sorts out the nonlinear issue of the fuel stack network by utilizing the high number of iterations. As a result, there is a possibility that the proposed fuel power generation system size and development complexity will be increased. So, the continuous different slope value-based cuckoo search method is hybridized along with the conventional power point identifiers for optimizing the iteration number of the proposed MPPT method.

## Data Availability

The datasets used and/or analyzed during the current study are available from the corresponding author upon reasonable request.
